# A Portable Immunosensor with Differential Pressure Gauges Readout for Alpha Fetoprotein Detection

**DOI:** 10.1038/srep45343

**Published:** 2017-03-24

**Authors:** Qingping Wang, Rongjie Li, Kang Shao, Yue Lin, Weiqiang Yang, Longhua Guo, Bin Qiu, Zhenyu Lin, Guonan Chen

**Affiliations:** 1 College of Environmental Science and Engineering, Fujian Normal University, Fuzhou, Fujian, 350007, China; 2 College of Chemistry and Chemical Engineering, Fujian Normal University, Fuzhou, Fujian, 350007, China; 3 Department of Thoracis Surgery, Cancer Hospital & Institute, CAMS & PUMC, Beijing, 100021, China; 4 MOE Key Laboratory of Analysis and Detection for Food Safety, Fujian Provincial Key Laboratory of Analysis and Detection Technology for Food Safety, College of Chemistry, Fuzhou University, Fuzhou, Fujian, 350116, China

## Abstract

A portable, affordable and simple detector is requested in a “Point-of-Care-Testing” (POCT) system. In this study, we exploited the potentialities of Differential Pressure Gauge (DPG) to the orientation of POCT technology. Alpha fetoprotein (AFP) was chosen as a model analyte that could specifically recognized by its antigen, and a tiny outfits equipped with a DPG was employed as the signal readout. Pt/SiO_2_ nanospheres were synthesized and modified with the detection antibody. In the presence of target, a sandwich of immunocomplex specifically formed and the Pt/SiO_2_ had been modified on the capture antibody. Which then can be dissolved to release plenty of Pt and the suspensions were transferred into a closed vial filled with appropriated amount of hydrogen peroxide. Subsequently, hydrogen peroxide was decomposed to produce oxygen, resulting in the enhancement of pressure in the closed vial and which can be detected by DPG easily. Under the optimized conditions, the read out signal from DPG had a direct relationship with AFP concentrations in the range of 10~200 ng/mL, and the detection limit was as low as 3.4 ng/mL. The proposed portable sensor had been successfully applied to detect AFP in serum samples with satisfactory results. This strategy holds a great promising in biological analysis as its convenient operations, reliable results and flexible apparatus.

Developing the technology of point-of-care testing (POCT) is meaningful for improving healthcare, maintaining sanitation and ensuring food safety due to the characteristic of “pocket” size, cost effectiveness, reliable quantitative results and easy operation^1^,^2^,^3^,^4^. Nowadays, more and more portable devices have been widespread used in daily life for analysis^5^, such as thermometer gauges for temperature, hygrometers for humidity, sphygmomanometer for blood pressure, personal glucose meter (PGM) for glucose and so on. In order to extend the application of portable devices, some groups have attempted to ameliorate them to be signal readout^6^. For examples, a common thermometer can related with ELISA to fabricate a biomolecular quantitation method based on the nanoparticle-mediated photothermal effect^7^; a portable infrared thermometer basing on an infrared detector has been commercialized, and a portable alcohol detector is widely used for alcohol testing based on a multimeter. The representative case is the personal glucose meter (PGM), used by diabetic patients themselves to monitor the concentration of blood glucose, has been transformed into a component of a sensor combining with biochemical techniques for quantifying non-glucose targets, such as DNA, enzyme and proteins^8^.

However, most of portable devices can only provide one signal which corresponds to a change of physical parameter and thereby restricted the application of those portable devices. In this study, we report a strategy exploiting the potentialities of Differential Pressure Gauge (DPG) to the orientation of POCT technology. In fact, pressure gauge is a useful device but has not been taken full advantages. Many chemical or biological reactions accompanied with a change of pressure, which can act as a signal reflecting the specific situation^9^,^10^. In hospital, a pressure gauge or a flow meter is used to monitor the service condition of an oxygen cylinder. Besides, a pressure gauge is essential to a pneumatic tire. However, little research about the detection of biochemical targets based on the pressure change has ever been reported. The main obstacle may stem from that the pressure change induced by biomedical targets is comparatively weak. Some groups attempted to design a capillary tube or volumetric bar-chart chip with a floating ink for indicating the micro-pressure change, which was caused by the decomposition of hydrogen peroxide to generate oxygen in a closed container. However, using the height of ink in channel as the data readout is apparently short of veracity and repeatability, because the results were susceptible to the device position located, the apparatus configuration designed and the individual proficiency of operations^11^,^12^. Furthermore, results just recorded on the paper were difficult to connecting with digital devices (i.e. iPads, computers, or phones with appropriate application programs), which emerged their increasing importance in personal health care^13^. In an early study, our group successfully employed a commercially available differential pressure gauge to set up an analytic method for thrombin detection^13^. However, it was found that catalase for catalysing the decomposition of hydrogen peroxide was inefficient and easily inactivated. It is necessary to find some way to address this problem.

Enzyme-linked immunosorbent assay (ELISA) is an important molecular recognition technology, which has been substantially used for biomarker quantification with high specificity^14^,^15^,^16^,^17^,^18^,^19^,^20^,^21^,^22^. In mostly of these biosensors, the signal readout relies heavily on optical-based measurements, which request measuring instrument such as ultraviolet spectrophotometer or fluorospectrophotometer. This is so inconvenient in on-pot determination or resource limited area. Though colorimetric immunoassays was proposed to be a simple, sensitive, and fast approach, it was still difficult to realize quantitative analysis since the naked eyes is insensitive to the slight color changing. Recently, ELISA had been coupled with glucose meter to develop new sensing systems for multiple bio-molecules detection. For examples, Yang group had reported a method that integrated ELISA with magnetic mesoporous silica and used glucose meter as signal readout for PbTx-2 detection^23^. Liu group combined ELISA and ring-oven washing technique for cancer biomarker detection^24^. But to the best of our knowledge, little literature using DPG as readout had been reported. Only Yang group currently had attempted this combined technique^25^. In theory, the decomposition of 1 mmol H_2_O_2_ generates 11.2 mL oxygen gas under standard conditions, which increases the pressure as high as 2.27 × 10^5^ Pa in a closed container of 10 mL, and these change caused by a trace of target would be detected by DPG easily. Therefore, it holds a great promise in combining ELISA with DPG to develop a POCT sensor for a fast, accurate and reliable assay.

As mentioned early, there were some drawbacks about the decomposition of H_2_O_2_ by the catalase. It has been reported that the present of Pt can decompose H_2_O_2_ with high efficiency and this reaction is inert to surrounding environments^26^. In this study, an immunosorbent-based assay equipped with DPG as signal readout has been developed for AFP determination; Pt has been embed in the SiO_2_ to prepare Pt/SiO_2_ nanoparticles firstly, which then be further modified with antibody through its amino groups on the surface. Then which has been used as the catalyst to decompose H_2_O_2_ to produce oxygen gas in a closed vial and causing the increasing of pressure in the vial, which can be used to present the AFP concentration. The proposed biosensor has been applied to detect AFP in serum samples with satisfied results.

## Methods

### Materials and Instruments

A commercially available differential pressure gauge (DPG) was purchased from BENETECH (GM511). Fourier Transform Infrared (FT-IR) spectra were recorded on a Nicolet 6700 (Thermo Fisher Scientific, USA) spectrometer in transmission mode. The High-Resolution Transmission Electron Microscopy (TEM) images were obtained with Tecnai G2 F20 S-TWIN TEM (FEI, USA).

Tetraethylorthosilicate (TEOS), 3-aminopropyltriethoxysilane (APTES) were purchased from Sigma-Aldrich. Hexadecyltrimethyl ammonium bromide (CTAB), Poly (vinylpyrrolidone) (PVP) (Mw = 29000), Dihydrogen hexachloroplatinate (H_2_PtCl_6_■6H_2_O) were purchased from Sinopharm Chemical Reagent (Shanghai, China), a Diagnostic Kit for Alpha fetal protein (ELISA) was purchased from Biocell Biotechnology (Zhengzhou China).

Mouse-derived antihuman antibody pairs for capturing AFP and human AFP antigen were obtained from BiosPacific (Emeryville, CA) and severed as detection antibody.

### Synthesis of Silicon dioxide Nanospheres

Amine-group functionalized silicon dioxide nanospheres were firstly synthesized by hydrothermally method according to the early reports^27^,^28^. Briefly, 0.25 g CTAB, 0.08 g NaOH was dissolved in water to a volume of 120 mL and heat to 353 K. Then 2.5 mL TEOS was drop wised to the surfactant solution followed by 245 μL APTES. After stirring for 2 h to give rise white precipitates, the solid product was filtered, washed three times with deionized water and methanol to completely remove CTAB and excessive MPTMS, then dried in a vacuum oven overnight.

### Doping Silica Nanospheres with Pt (Pt/SiO_2_)

The loading of Pt into silica nanospheres was performed by refluxing a mixture of 0.5 g silica nanospheres, 26.6 mg PVP and 124.3 mg H_2_PtCl_6_●6H_2_O in 400 mL water/methanol solution for 3 h. It was found that the color of the mixture was turn into black from pale yellow^29^. Then the solution was cooled down to room temperature slowly, and centrifuged at 9000 rpm for 7 min. The precipitates were washed three times with deionized water to get the Pt doping silica nanospheres functionalized with amine-group (Pt/SiO_2_).

### Modification of Pt/SiO_2_ with Detection Antibody

The as-synthesized Pt/SiO_2_ nanoparticle was labelled with detection antibody by cross-linker of glutaraldehyde. 1.5 mg of Pt/SiO_2_ was dissolved into 1 mL sodium bicarbonate buffer (100 mM, pH 8). Then 1 mL 5% glutaraldehyde stock solution (w/v) was added and slight stirring for 30 min at room temperature. The mixture was centrifuged at 9000 rpm to remove excess glutaraldehyde, and the nanoparticles were re-suspended in 2 mL PBS (100 mM, pH 7.3). Next, 50 μL detection antibody of 10 mg/mL was added to the suspension drop by drop with stirring. After incubation at room temperature for 1 h, the suspension was centrifuged at 9000 rpm to remove the excess antibody. At last, the unsaturated binding was blocked by bovine serum albumin (BSA) (10% w/v in deionized water). The detection antibody functionalized Pt/SiO_2_ was re-suspended in PBS and stored at 4 °C for further usage.

### Procedures of Immunosorbent Assay with DPG

Firstly, 100 μL at various concentrations (10~200 ng/ml) of AFP antigen solution with BSA blocking were added into the 96-wells get from the diagnostic kit and incubated on a shaker (500 rpm) for 30 min. Then the plate was drained and washed by wash buffer (100 mM PBS with 0.05% Tweeen-20) three times. After that, 100 μL detection antibody functionalized Pt/SiO_2_ was added into each well, sealed and incubated at room temperature on a shaker (500 rpm), washed three times by washing buffer and tapped dry. Finally, the Pt/SiO_2_ was dissolved by 100 μLNaOH (0.10 M) and incubated on a shaker (100 rpm) for 10 min, 100 μL of HCl (0.01 M) was added to the well to regulate the pH value. Then the released Pt was collected and injected into a tiny outfit fabricated by ourselves. And the pressure in the outfit was monitored by the DPG.

## Results and Discussion

### Principle of the portable sensor for AFP

**Figure 1:**
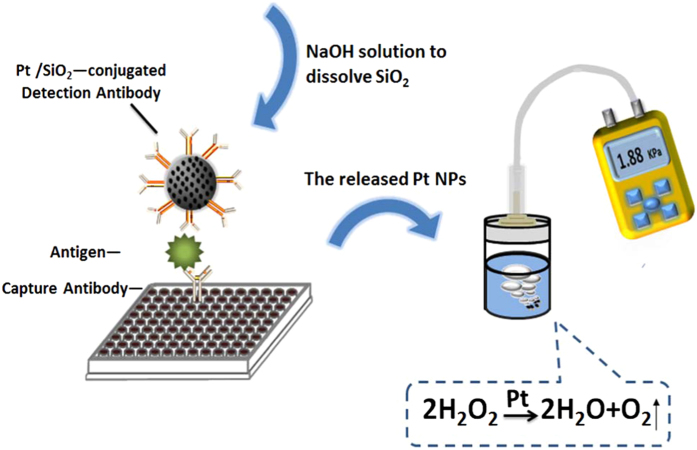
Principle of the immunesorbent-based sensor with a DPG as readout.

Figure 1 shows the details of the proposed approach for AFP detection, which consists of the imunosorbent-based target recognition and the signal-converted micro-pressure monitoring by a tiny outfits fabricated by ourselves. As the linker of the two parts, silica nanospheres doped with Pt (Pt/SiO_2_) was firstly synthesized and then modified on AFP antibody. In the presence of AFP, a sandwich-liked immunocomplex of detection antibody-AFP-capture antibody (coated in the well) specifically formed. After removing the excess detection antibody, the immunocomplex was destroyed by the addition of NaOH solution to dissolve SiO_2_, resulting in a plenty of released Pt. The suspensions containing Pt were subsequently transferred into a vial filled with 4 mL H_2_O_2_. It was found that a lot of bubble generated since the H_2_O_2_ was decomposed to H_2_O and O_2_ by Pt nanoparticles. And the enhancement of pressure in an airtight vial caused by the accumulation of O_2_ was recorded by DPG during the whole process. Since the pressure detected has a relationship with the AFP concentration, a simple portable biosensor for AFP detection can be developed.

### Characterizations of the Pt/SiO_2_

**Figure 2:**
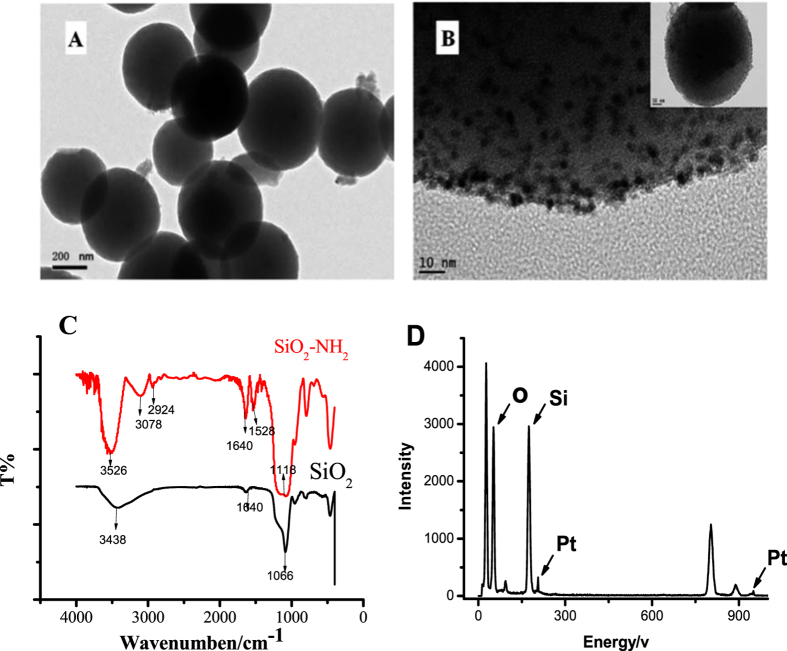
(**A**) TEM images of SiO_2_; (**B**) High-resolution TEM images of Pt nanoparticles grown on SiO_2_ (Pt/SiO_2_); (**C**) FTIR spectrum of Amine-group functionalized Silica Nanospheres with Pt nanoparticles loading, and (**D**) the corresponding Energy-dispersive X-ray spectroscopy.

To deposit Pt nanoparticles directly on the surface of SiO_2_, a template strategy was employed here according to the early reported method^30^,^31^. Primary amine was found to be an effective functional group to combine with metal ions. So the NH_2_- SiO_2_ can promote the reduction of Pt^4+^ located on the surface of SiO_2_ to produce Pt nanoparticles. High-Resolution Transmission Electron Microscopy has been applied to characterize the morphology of SiO_2_ and Pt/SiO_2_. As shown in Fig. 2(A), the diameter of SiO_2_ was about 400 nm. And high density of Pt nanoparticles with diameter of 2 nm were observed on the surface of the SiO_2_ (Fig. 2(B)), which confirmed that Pt had loaded in the silicon nanospheres effectively.

Fourier transform infrared (FTIR) spectroscopy of the Pt/SiO_2_ was also been tested. Although the stretching vibration peak of –NH_2_ is overlapped with –OH (3100~3600 cm^−1^), and the deformation vibration peak of –NH_2_ is overlapped with characteristic peak of Si-O-Si. (15600~1650 cm^−1^). The peaks appear at 3078, 2924, 1528 cm^−1^ is the characteristic peaks of C-H in APTES. (Figure 2(C)) These evidences indicated that amine groups had been modified the silica nanospheres. And energy-dispersive X-ray spectroscopy (EDX) had been used to further confirm the presence of platinum in the silicon nanospheres. As shown in Fig. 2(D), it was found that the Pt characteristic peak and the percentage of platinum was calculated to be about 5.3%.

### Optimized of the reaction conditions

**Figure 3:**
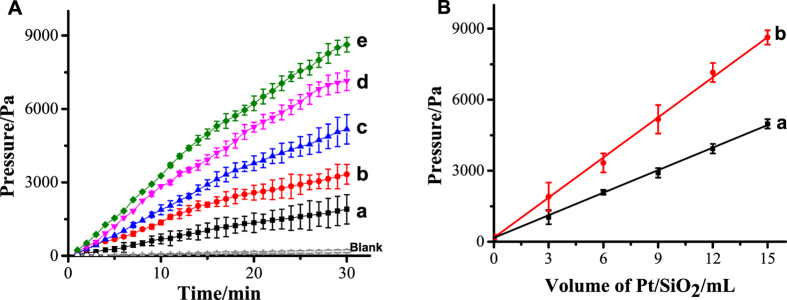
(**A**) Time-dependent changes in pressure with different volume of Pt/SiO_2_ (1.00 mg/mL):Blank 0, (a) 3, (b) 6, (c) 9, (d) 12,(e) 15 μL in the tiny outfit containing 4 mL of 1.6 mol●L^−1^ H_2_O_2_. (**B**): the linear relationship between pressure signal and the volume of Pt/SiO_2_ at different reaction time, 15 min (a) and 30 min (b).

First of all, in order to verify the feasibility of our tiny outfits on the detection of micro-pressure change, Pt/SiO_2_ without any modifications was chosen as the test sample. Figure 3(A) shows the time-dependent changes of pressure with different amount of Pt/SiO_2_. It is found that the pressure of the system linearly increased with the extension of the reaction time before 30 min. Accordingly, there has a linear relationship between pressure and the amount of Pt/SiO_2_, 15 min (a) and 30 min (b), respectively (shown in Fig. 3(B)). These results indicate that there has relationship between the detected pressure and the amount of Pt/SiO_2_, and we may change the reaction time to meet the requirement of the detection limit. In this study, 15 min was selected as the reaction time without special mentioned.

**Figure 4:**
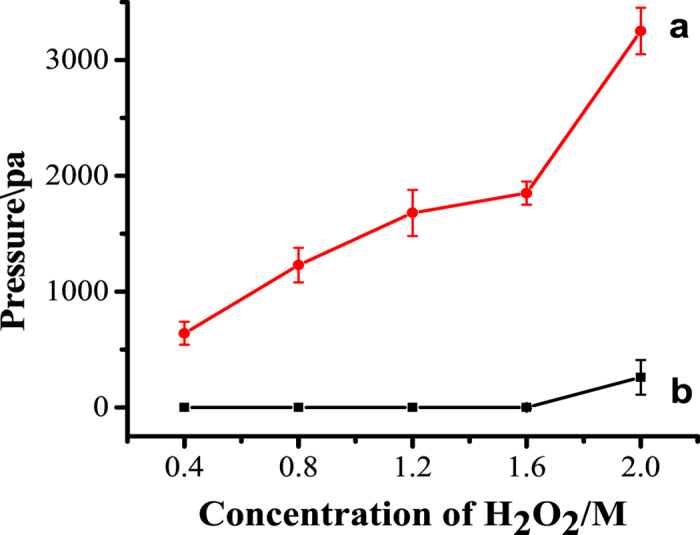
Pressure of different concentration of H_2_O_2_ decomposition reaction with 5 μL of Pt/SiO_2_ (red curve) with the procedure same as the procedure of AFP detection, and the corresponding backgrounds (black curve).

The concentration of H_2_O_2_ is another key role in this detection system. As shown in Fig. 4, with the increasing concentration of the H_2_O_2_ from 0.4 to 1.6 M (4 mL), the trend of pressure increases tardily, while a sharply increases is observed when the concentration of H_2_O_2_ is 2.0 M (the red curve). The reason for this phenomenon mainly stems from the unstable feature of H_2_O_2_, which implies a higher concentration of H_2_O_2_ over 1.6 M would decompose automatically and cause corresponding background. Thus, 1.6 M was chosen as the suitable concentration of H_2_O_2_.

### Quantificational detection of AFP

**Figure 5:**
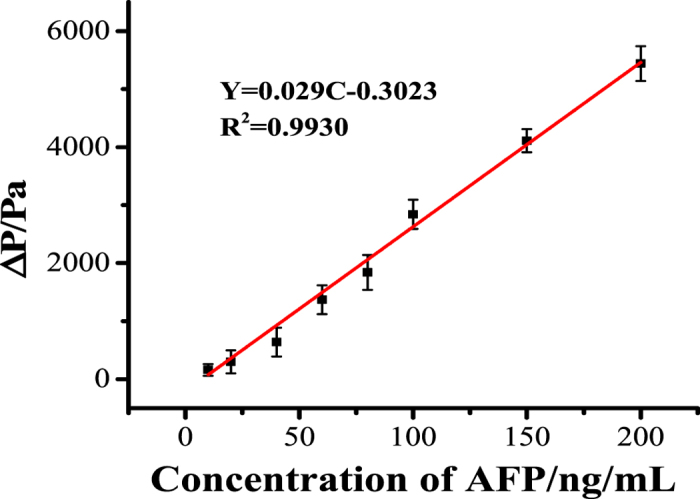
Relationship between the enhanced pressure and the concentration of AFP.

Figure 5 shows the relationship between the pressure detected and the AFP concentration. The pressured signal recorded by DGP increased with the increasing of AFP concentration. And there is a good linear relationship between the enhanced signal and the concentration of AFP in linear range of 10~200 ng/mL, the regression equation can be expressed as follow:


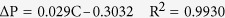


where ΔP is the difference of pressure signal recorded by PDG at present and absent of target, C is the concentration of AFP, while R is the regression coefficient. The detection limit is calculated to be 3.4 ng/mL (S/N = 3). This result meets the need of practical application since the average level of AFP in healthy human serum is lesser than 20 ng/mL.

It should be noted that although some conventional ELISA method gives out a lower detection limit, it is actually difficult to distinguish the subtle change in color only by naked eye without any assistant of instruments. As regards of the portable assay, DPG equipped ELISA assay provides a promising approach for point-of-care testing with an accurate and reliable result. Besides, there are many strategies characterized by portable or rapid detection also show deficiency in LOD, Table 1 shows several examples.

### Selectivity of the sensor

**Figure 6:**
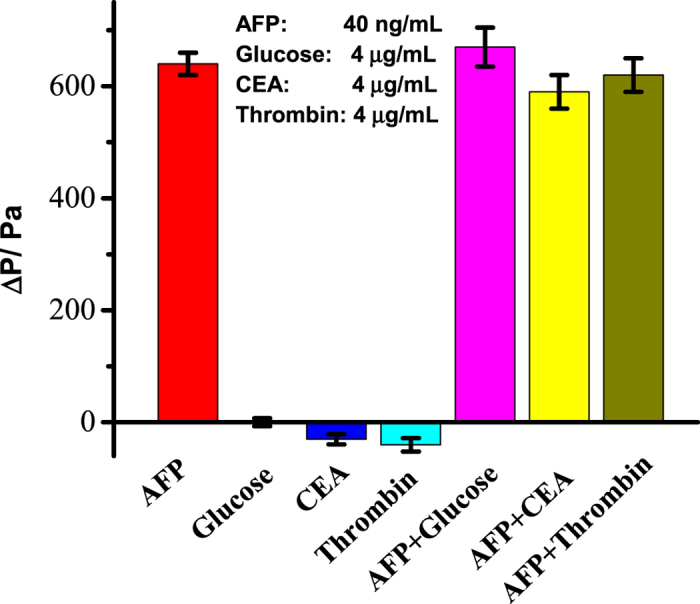
Selectivity of the sensor for AFP detection.

The specificity of this assay for AFP detection was investigated by testing several interferences that commonly meet during the detection of AFP, such as glucose, thrombin and CEA. The concentration of AFP was fixed at 40 ng/mL while the other interferences were all 100 folds. Experiments were conducted under the same conditions and repeated three times for each situation. As shown in Fig. 6, the pressure enhanced greatly at present of target but nearly no signal changing had been detected if we added interferences only. These results clearly demonstrated the high specificity of the developed assay.

### Serum sample detection

The proposed biosensor had been applied to detect the AFP concentration in the real samples (Three volunteers from the first affiliated hospital of Fujian Medical University). As shown in Table 2, the results detected by the proposed method had good agreements with the reference values provided by the hospital. Different levels of AFP had been added into the samples and were tested by the presented method, and results showed that recoveries of AFP were in the range of 96.6~103.3% with RSDs of 1.2~4.3%. These indicate the proposed method can be applied to detect AFP in the clinic samples with satisfactory results.

## Conclusions

In summary, an immunesorbent-based assay using PDG as signal readout for AFP detection is developed. Pt/SiO_2_ nanoparticles were used as nanotags modified with detection antibody, as well as the catalyst of H_2_O_2_ solution to produce O_2_. In the presence of AFP, a sandwich immune complex of capture antibody-AFP-detection antibody specifically formed. After destroying the Pt/SiO_2_ nanospheres, the plenty of released Pt nanoparticles were subsequently transferred into an airtight vial. In consequence, the target-induced pressure change can be detected by DPG easily and give out a fine quantificational result. This presented approach provides a reliable option to quantitative analysis of AFP with good selectivity. By alter the corresponding antigen-antiboby, this assay would be extend to any bioassay using a catalyst as tags. What’s more, the features of pocket-size, low-cost and wide potential applications in biological analysis may become the new highlight of PDG.

## Additional Information

**How to cite this article**: Wang, Q. *et al*. A Portable Immunosensor with Differential Pressure Gauges Readout for Alpha Fetoprotein Detection. *Sci. Rep.* **7**, 45343; doi: 10.1038/srep45343 (2017).

**Publisher's note:** Springer Nature remains neutral with regard to jurisdictional claims in published maps and institutional affiliations.

## Figures and Tables

**Table 1:** Comparison of the current method with other portable or rapid detection strategies.

Methodology	LOD	Instrumentation	testing time	Advantage	Refs
ELISA	3.4 ng/mL	DPG	15 min	Rapid and portable	This work
Microfluidic	12.5 ng/mL	Microfluidic chip	20 min	Rapid	1
Electrochemical	6 ng/mL	electrochemical workstation	5 min	Rapid	2
ELISA and LSPR	24 ng/mL	UV–vis–NIR	25 min	portable	3
CPs	69 ng/mL	XRF	2 min	convenient and cost-effective	4

**Table 2:** Determination of AFP in serum samples.

Sample	Reference value (ng/mL)	Detected value (ng/mL)	Add (ng/mL)	Total detected value (ng/mL)	Recovery (%)	RSD (%)
Serum 1	19.50	19.73	20.00	39.04	96.6	3.7
			30.00	59.64	99.5	2.0
			40.00	60.38	103.3	4.3
Serum 2	34.85	34.65	20.00	55.02	101.1	2.8
			30.00	64.91	100.2	1.4
			40.00	74.60	99.86	1.2
Serum 3	40.93	41.11	20.00	60.57	98.67	2.7
			30.00	70.03	97.4	1.6
			40.00	81.46	100.9	3.4
